# Interleukin-21 Enhances Rituximab Activity in a Cynomolgus Monkey Model of B Cell Depletion and in Mouse B Cell Lymphoma Models

**DOI:** 10.1371/journal.pone.0067256

**Published:** 2013-06-25

**Authors:** Cecile M. Krejsa, Rick D. Holly, Mark Heipel, Ken M. Bannink, Rebecca Johnson, Richard Roque, Jane Heffernan, Julie Hill, Lay Chin, Felecia Wagener, Faith Shiota, Katherine Henderson, Pallavur V. Sivakumar, Hong-Ping Ren, Fariba Barahmand-pour, Don Foster, Chris Clegg, Wayne Kindsvogel, Rafael Ponce, Steven D. Hughes, Kim Waggie

**Affiliations:** 1 Department of Pre-clinical Development, ZymoGenetics, Incorporated, a Bristol-Myers Squibb Company, Seattle, Washington, United States of America; 2 Department of Research, ZymoGenetics, Incorporated, a Bristol-Myers Squibb Company, Seattle, Washington, United States of America; The Scripps Research Institute and Sorrento Therapeutics, Inc., United States of America

## Abstract

Rituximab, a monoclonal antibody targeting CD20 on B cells, is currently used to treat many subtypes of B cell lymphomas. However, treatment is not curative and response rates are variable. Recombinant interleukin-21 (rIL-21) is a cytokine that enhances immune effector function and affects both primary and transformed B cell differentiation. We hypothesized that the combination of rIL-21 plus rituximab would be a more efficacious treatment for B cell malignancies than rituximab alone. We cultured human and cynomolgus monkey NK cells with rIL-21 and found that their activity was increased and proteins associated with antibody dependent cytotoxicity were up-regulated. Studies in cynomolgus monkeys modeled the effects of rIL-21 on rituximab activity against CD20 B cells. In these studies, rIL-21 activated innate immune effectors, increased ADCC and mobilized B cells into peripheral blood. When rIL-21 was combined with rituximab, deeper and more durable B cell depletion was observed. In another series of experiments, IL-21 was shown to have direct antiproliferative activity against a subset of human lymphoma cell lines, and combination of murine IL-21 with rituximab yielded significant survival benefits over either agent alone in xenogeneic mouse tumor models of disseminated lymphoma. Therefore, our results do suggest that the therapeutic efficacy of rituximab may be improved when used in combination with rIL-21.

## Introduction

Interleukin-21 (IL-21) is a class I cytokine produced by CD4+ and NK-T cells that acts directly on B cells, T cells, monocytes, dendritic cells and natural killer (NK) cells. It signals via STAT phosophorylation through a heterodimeric receptor comprised of the IL-21 receptor (IL-21R) and the common gamma chain (γ_C,_ CD132) [1], [2]. Actions of IL-21 on human [3], [4] and mouse [4], [5], [6] B cells have been extensively studied. IL-21 has been reported to promote B cell differentiation and, depending on the stimulation context, may inhibit B cell proliferation. Recently, the IL-21R has been found on primary cells and cell lines from diffuse large B cell and follicular cell lymphomas, and on primary mantle cell lymphoma and chronic lymphocytic leukemia cells. *In vitro* IL-21 treatment of cells from these tumors induced STAT signaling and was associated with growth arrest and tumor cell apoptosis [7], [8].

The anti-tumor activity of IL-21 has also been demonstrated in several mouse tumor models, and was found to be partially or completely dependent upon NK cells and/or CD8 T cells [9], [10], [11]. IL-21 has been evaluated as a monotherapy in early clinical trials for treatment of metastatic melanoma and renal cell carcinoma, and as a combination therapy with rituximab for non-Hodgkin B cell lymphomas (NHL) [12], [13].

Rituximab is a chimeric monoclonal antibody (mAb) with high binding affinity for human and cynomolgus monkey CD20, a trans-membrane B cell differentiation antigen. The exact mechanism of rituximab action has not been determined; however, it is thought to include induction of apoptosis following CD20 engagement, complement dependent lysis, and Fc-mediated killing of CD20+ B cells by effector cells [14], [15]. Rituximab monotherapy is effective in treating indolent B cell NHL; however, the mean duration of response is about 12 months and approximately half of patients do not respond [16], [17]. Further manipulation of effector cell function to improve patient response and survival rates has been the basis for clinical trials combining rituximab with IL-2 [18] or IL-12 [19]. Results of preclinical studies presented here show that IL-21 is also an attractive candidate for combination with rituximab to treat B cell lymphomas.

## Methods

Blood samples were procured from an in-house volunteer donor program at ZymoGenetics. Donors were screened for blood-borne pathogens prior to acceptance into the program and signed a written consent form. The samples were anonymized prior to them being given to the investigator so that the sample could not be associated with a specific donor.

Animal studies were carried out in accordance with the recommendations in the Guide for the Care and Use of Laboratory Animals (National Research Council). Murine protocols were approved by the ZymoGenetics Institutional Animal Care and Use Committee (Protocol Number 016). Nonhuman primate protocols were conducted at contract research organizations (Covance Laboratories GmbH, Munster, Germany, Study Number 2209-007, approved by the Landesamt für Natur, Umwelt, und Verbraucherschutz (LANUV) Nordhein-Westfalen) and SNBL USA, Everett, WA (Study Number 002.13, approved by the SNBL USA Institutional Animal Care and Use Committee) in compliance with applicable laws, regulations, and guidances.

### Reagents and Cell Lines

Recombinant human IL-21 (rIL-21) was expressed in *E. coli* inclusion bodies at ZymoGenetics as the N-terminal methionylated form of the molecule. Following isolation and washing of the inclusion bodies, rIL-21 was solubilized, refolded and purified using cation exchange chromatography and hydrophobic interaction chromatography. The purified rIL-21 was buffer exchanged into formulation buffer and stored frozen. Prior to use in these studies the rIL-21 was thawed and diluted to the target concentration with 0.9% sodium chloride (NaCl) for injection. Recombinant mouse IL-21 (mIL-21) was produced in *E. coli* at ZymoGenetics, and purified per internal protocols. The mIL-21 was stored frozen, and then thawed and diluted with 0.9% NaCl immediately prior to administration.

Rituximab for injection (Genentech, South San Francisco, CA) was provided at 10 mg/mL from the pharmacy; stability and formulation information are in the product label. Rituximab was diluted with 0.9% NaCl prior to administration.

Monoclonal antibody (mAb) clone information for flow cytometry studies is provided in Table 1.

**Table 1:** Monoclonal antibodies used in flow cytometry.

Antigen	Clone	Supplier
CD3	Sp34, Sp34-2	BD^1^
CD4	RPA-T4	BD
CD8	RPA-T8, 3B5	BD, Caltag^2^
CD11b	ICRF44	BD
CD11c	S-HCL-3	BD
CD14	M5E2	BD
CD16	3G8	BD
CD19	HIB19	BD
CD20	2H7, L27	BD
CD21	B-ly4	BD
CD25	M-A251	BD
CD27	M-T271	BD
CD23	9P25 (ML233)	Coulter^3^
CD32	FLI8.26	BD
CD40	5C3	BD
CD64	22	Coulter
CD80	L307.4	BD
CD122	Mik-B2	BD
CD138	MI15	BD
HLA-DR	G46-6	BD
IgD	IA6-2	BD
IgM	G20-127	BD
Granzyme B	GB12	Caltag^2^
NKG2D	149810	R&D Systems^4^
Perforin	dG9	BD
ICAM-1	HA58	BD
IgD	IA6-2	BD
CD56	My31	BD

1 BD Biosciences, Franklin Lakes, NJ.

2 Caltag Laboratories, Burlingame, CA.

3 Beckman Coulter, Brea, CA.

4 R&D Systems, Minneapolis, MN.

Anti-IL-21R mAb, a mouse IgG2a, kappa antibody reactive against the extracellular domain of human IL-21R, was generated against the heterodimeric Fc fusion protein IL-21R/γc-Fc at ZymoGenetics and biotinylated for flow cytometry analyses. Phycoerythrin-labeled streptavidin was used to detect IL-21R mAb staining. Trastuzumab for injection (Genentech) was obtained from the pharmacy.

Human B lymphoma cell lines included WSU-NHL and DOHH2 (DSMZ, Braunschweig, Germany) and MC116, IM-9, CESS, Raji, Ramos, Namalwa and HS-Sultan (ATCC, Rockville, MD). All lymphoma lines were grown in conditions recommended by the supplier.

### Human and Monkey ADCC Assays

Human NK cells were purified from peripheral blood mononuclear cells (PBMC) isolated from healthy donors using a Human NK Cell Kit (STEMCELL Technologies, Vancouver, B.C.), and cultured 3 days in 25 ng/mL rIL-21 or control media. Target cells were labeled with Calcein-AM (Life Technologies Corporation, Grand Island, NY) prior to incubation with NK cells plated at varying effector to target (E:T) ratios in HBSS containing 2 µg/mL rituximab or trastuzumab, human IgG (Jackson ImmunoResearch, West Grove, PA), or no antibody. For specific target cell lysis the fluorescence at 517 nm of calcein released into the media was calculated according to the formula: Percentage lysis = (ADCC lysis-nonspecific lysis/total lysis-nonspecific lysis) × 100.

The cynomolgus monkey assay used PBMC isolated from 8 male cynomolgus monkeys as effector cells. The number of NK cells was assessed by flow cytometry. ADCC against trastuzumab-coated BT-474 cells was assayed after culture in media alone or with 10 ng/mL rIL-21 for 24 to72 hours. Some studies used fresh PBMC preparations, i.e. without culturing prior to the ADCC analysis.

### Flow Cytometry

Antibody reagents were confirmed to cross-react with appropriate cell surface antigens in cynomolgus monkeys (Table 1). Whole blood samples from human or cynomolgus monkeys were stained with fluorochrome-conjugated antibodies or isotype controls; erythrocytes were lysed and samples fixed prior to analysis. For intracellular proteins, human NK cells or monkey PBMCs were stained with surface markers, then washed and treated with Cytofix/Cytoperm buffer (BD Biosciences, Franklin Lakes, NJ). Cells were stained with Granzyme B-, perforin-, phospho-STAT-specific, or isotype control antibodies diluted to 1∶400 in Perm/Wash buffer, and analyzed on a FacsCalibur (BD Biosciences) or FacsAria (BD Biosciences) cytometer. For absolute counts, percentage data from flow cytometry analyses were converted using matched hematology samples.

### Cynomolgus Monkey Studies

Two cynomolgus monkey studies were conducted. The monkeys were either individually (first study) or pair-housed (second study) and provided with enrichment toys such as stainless steel mirrors, plastic tools, and plastic balls. The monkeys were given water ad libitum. Biscuits (Purina Mills Laboratory Fiber Plus Monkey Diet, Animal Specialties, Hubbard, OR or Ssniff Pro, Ssniff Spezialdiaten GmbH, Soest, Germany) were given twice daily and supplemented with fruit and vegetable or bread treats. In order to mimic the clinical setting and better observe the monkeys for adverse drug effects, dosing and blood sampling was conducted on nonanesthetized animals. The monkeys were observed at least twice daily during the course of the study. At the end of the study, the monkeys were sedated with ketamine hydrochloride given i.m., and then given an i.v. overdose of sodium pentabarbitone followed by exsanguination.

In the first study, 12 adult male, treatment-naïve cynomolgus monkeys were acclimated and randomized to study groups, 3 animals per group, and baseline values obtained for all in-life measurements (SNBL USA, Everett, WA). Two consecutive once-weekly rituximab doses (0.05 mg/kg, days 1 and 8) were given by slow intravenous (i.v.) injection, and a third dose was administered two weeks later (day 22). Dosing with rIL-21 (0.5 mg/kg) was by slow i.v. injection on days 1–3, 8–10 and 22–24 for a total of 9 injections. The study was terminated on day 42.

In a second study, twenty-four adult male, treatment-naïve cynomolgus monkeys were acclimated, randomized to study groups, and baseline samples obtained for all in-life measurements (Covance Laboratories, Münster Germany). All groups were dosed with rituximab (10 mg/kg) by i.v. infusion (200 mg/hr, 1 mL/min.) once-weekly for 4 weeks. Either rIL-21 (0.3 or 1.5 mg/kg) or vehicle (0.9% NaCl) was administered weekly by slow i.v. injection immediately after rituximab dosing. Four animals from each dose group were euthanized on day 24, and remaining animals were euthanized on day 52, following a dose-free period of 30 days. A full necropsy was conducted and a comprehensive panel of tissues was collected, processed and examined for gross and microscopic lesions by a veterinary pathologist. Selected immune tissues were processed for immunohistochemistry according to standard procedures.

### Lymphoma Growth Inhibition Assay

Duplicate 1 mL cultures were initiated with 5×10^4^ IM-9, MC116, WSU-WHL, Raji, CESS, HS-Sultan, DOHH2, or Ramos B cell lymphoma cells in RPMI 1640 media with or without rIL-21 (50 ng/mL). After 24, 48 and 72 hours, viable cell counts were performed. After 72 hours, 5×10^4^ viable cells were re-plated in 1 mL of the corresponding media, and counts were analyzed after 72, 96, and 120 hours of additional incubation. Two independent experiments were performed for each lymphoma line. The percentage change in cell counts in the rIL-21 treated cultures, compared to control cultures, was calculated on day 7.

### Xenogeneic Disseminated Lymphoma Models

Eight-to-10 week old female SCID (C.B-17/lcrHsd-*Prkcd^scid^*; Charles River Labs) or NOD/SCID (NOD/LtSz*^scid^*; Jackson Labs, Bar Harbor, ME) mice were used. The mouse colonies of origin were routinely tested and found to be free of common murine pathogens. Mice were maintained in ventilated microisolator units and had ad libitum access to filtered water and irradiated rodent chow (#5053, PicoLab, Richmond, IN).

Mice were injected i.v. with 1×10^6^ tumor cells (0.1 mL) on day 0. For monotherapy, rIL-21 was administered subcutaneously by mini-osmotic pumps (Model 2004, Alzet, Santa Clara, CA) on day 1. These pumps delivered 12 µg/day mIL-21 for 28 days based on their nominal infusion rate. The rituximab dose used for each lymphoma line, determined in pilot studies, provided a modest increase in median survival time. For combination therapy of HS-Sultan bearing mice, 100 µg mIL-21 was injected intraperitoneally (i.p.) on days 1–5 and 20 µg rituximab was injected i.p. on days 3, 7, 11, 15 and 19. For Raji-bearing mice, 100 µg mIL-21 was injected i.p. on days 3–7 and 20 µg rituximab was given i.p. on days 5, 9, 13, 17, and 21. To deplete neutrophils, SCID mice were injected i.p. with 50 µg of anti-Gr-1 polyclonal antibody (Wako Chemicals USA, Inc., Richmond, VA) on days –1, 4, 9 and 14, which resulted in a mean reduction of 94% in peripheral blood neutrophils in preliminary studies. To deplete macrophages, clodronate liposomes were prepared and the protocol for long-term depletion of macrophages was followed [20]. Clodronate liposomes or control PBS liposomes (containing 8.6 mg/ml phosphatidyl choline) were injected i.v.: 0.2 mL on day 3 and 0.1 mL on days 9, 15 and 21. This regime depleted 95% of F4/80+ cells from liver and 90% from the spleen in preliminary studies. Animals were observed daily and euthanized if hind limb paralysis or ≥20% weight loss was observed. Euthanasia was by means of isoflurane or carbon dioxide overdose followed by cervical dislocation.

### Statistical Analyses

For mouse lymphoma models, survival curves were plotted and compared using the LogRank Test and GraphPad PRISM 4 software (San Diego, CA). Data from *in vitro* assays were analyzed using Microsoft Excel and PRISM. Flow cytometry results were analyzed using CellQuest or FacsDiva software (BD Biosciences). The AUC analysis of monkey B cell dynamics used the trapezoidal method, and mixed effect models were generated using SAS (Cary, NC).

## Results

### *In vitro*, IL-21 Activates Innate Effector Mechanisms and Acts Directly on Human B Cells

**Figure 1:**
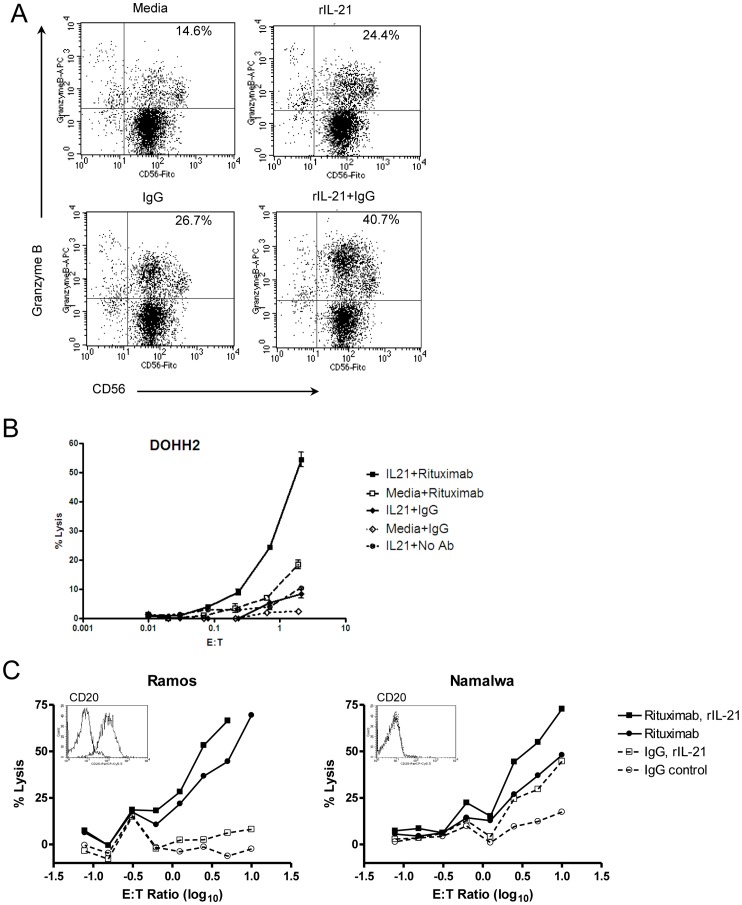
Treatment with rIL-21 enhances ADCC activity in human NK cells. (A) Induction of granzyme B in NK cells cultured with rIL-21, IgG or both. (B, C) Purified human NK cells were cultured with rIL-21 (25 ng/ml) or media for 3 days and then placed in ADCC assays with control IgG or rituximab (2 µg/ml). Percentage lysis in representative assays against DOHH2 targets (B), and lysis of Ramos cells compared to Namalwa cells (C) are shown. *Insets* show CD20 expression on target cell lines. Data shown are representative results from 2 separate co-culture experiments (A) and from separate experiments using 6 NK cell donors and 5 lymphoma lines (B, C). E:T = effector to target cell ratio.

Human and monkey NK cells were cultured with rIL-21 to specifically examine ADCC, using trastuzumab- or rituximab-coated tumor cells as targets. Co-culture of human NK cells with rIL-21 and human IgG increased both the percentage of cells expressing granzyme B and the staining intensity of positive cells (Figure 1A). No change in CD16 expression was observed on human NK cells. Human NK cells pretreated for 72 h with rIL-21 mediated a higher percentage of lysis of rituximab-coated DOHH2 lymphoma cells than of media-treated NK cells (Figure 1B). rIL-21 also enhanced ADCC for Namalwa cells, which express low levels of CD20, as well as Ramos cells, which highly express CD20 (Figure 1C) [21]. This was true for NK cells from all donors tested (n = 6).

**Figure 2:**
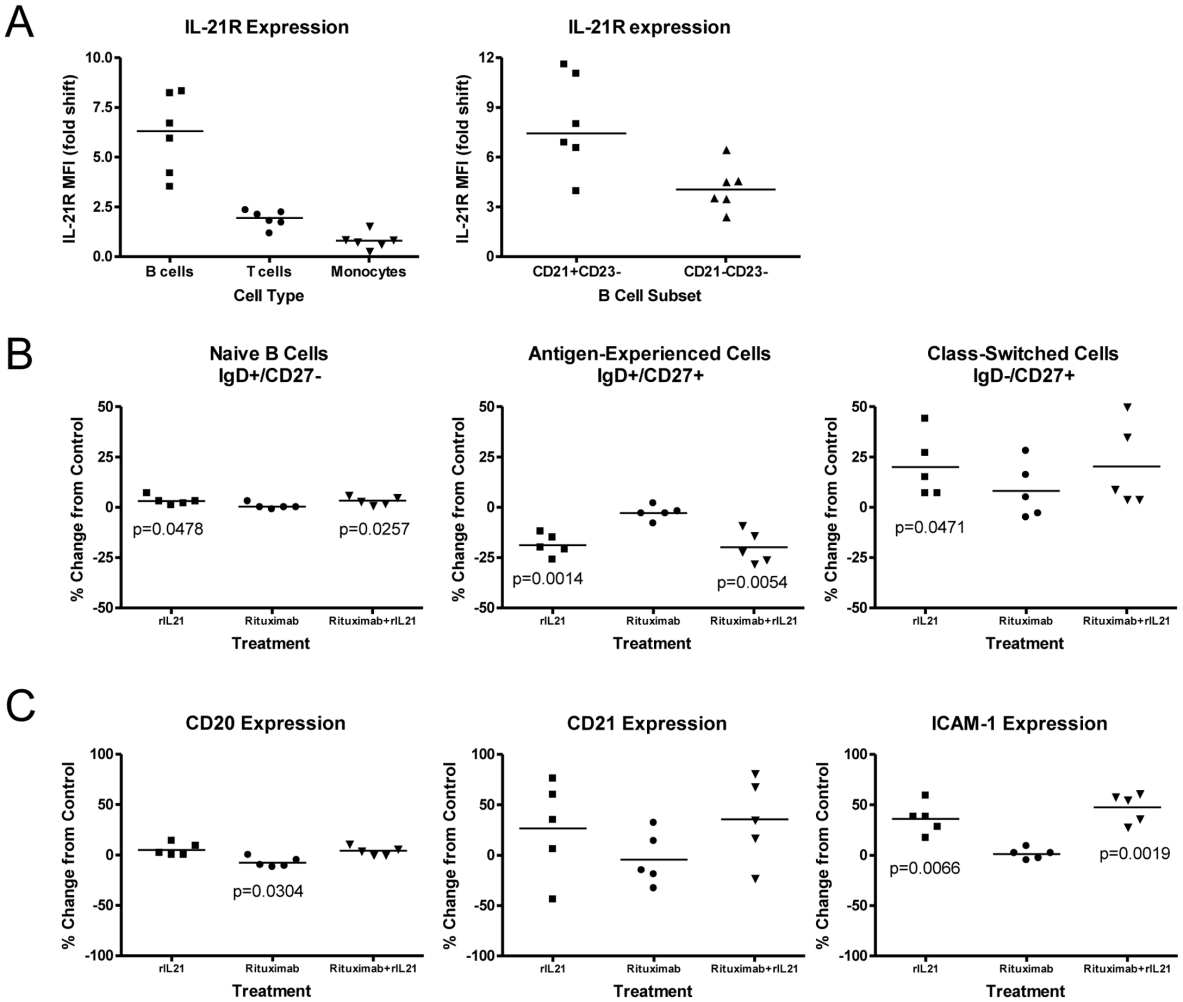
Effects of rIL-21 on normal human B cells. (A) IL-21R expression on B cells, T cells and monocytes. Median and individual mean fluorescence intensity (MFI) data from 5–6 donors are shown. Right panel shows higher expression of IL-21R on CD21+ B cell subset. (B, C) Effect of rIL-21 culture on B cell phenotype and expression of cell surface markers. Median percentage change from 48 hr control cultures, and significance levels of *t* tests are shown. (B) rIL-21 treatment shifts CD20+ B cells to class-switched phenotype. (C) Expression of surface markers following rituximab and rIL-21 cultures.

Normal primary human B cells showed higher expression of IL-21R than T cells or monocytes, with highest expression on the CD21+ subset of B cells (Figure 2A). *In vitro* treatment of B cells with rIL-21 did not result in proliferation or death, although a shift to the class-switched population was noted, with an average of 19% less cells in the antigen-experienced subset and a 20% increase in class-switched cells compared to the control group (Figure 2B). Culture with rIL-21 did not alter CD20 expression, whereas surface-phase rituximab did induce a small but significant (7.7%; p = 0.03) reduction in CD20 expression after 48 h culture (Figure 2C). Expression of CD21 was variably increased, depending on the donor, while ICAM-1 increased by an average of 36% and 47%, respectively following rIL-21 treatment alone or in combination with rituximab.

### IL-21 Enhances ADCC and Rituximab-mediated Depletion of B Cells in a Cynomolgus Monkey Model

Two studies to evaluate the *in vivo* effects of combining rIL-21 and rituximab were conducted in cynomolgus monkeys. In the first study, a rIL-21 dose (0.5 mg/kg) was chosen to provide strong activation of effector cells including NK cells, while a subclinical rituximab dose (0.05 mg/kg) was used to deplete only a fraction of B cells [22]. ADCC activity in PBMC preparations and flow cytometric evaluation of peripheral blood B cells and other leukocytes were assessed at various time-points. Trastuzumab-coated BT-474 cells were used in the ADCC assay to avoid potentially confounding effects from the *in vivo* rituximab treatment.

**Figure 3:**
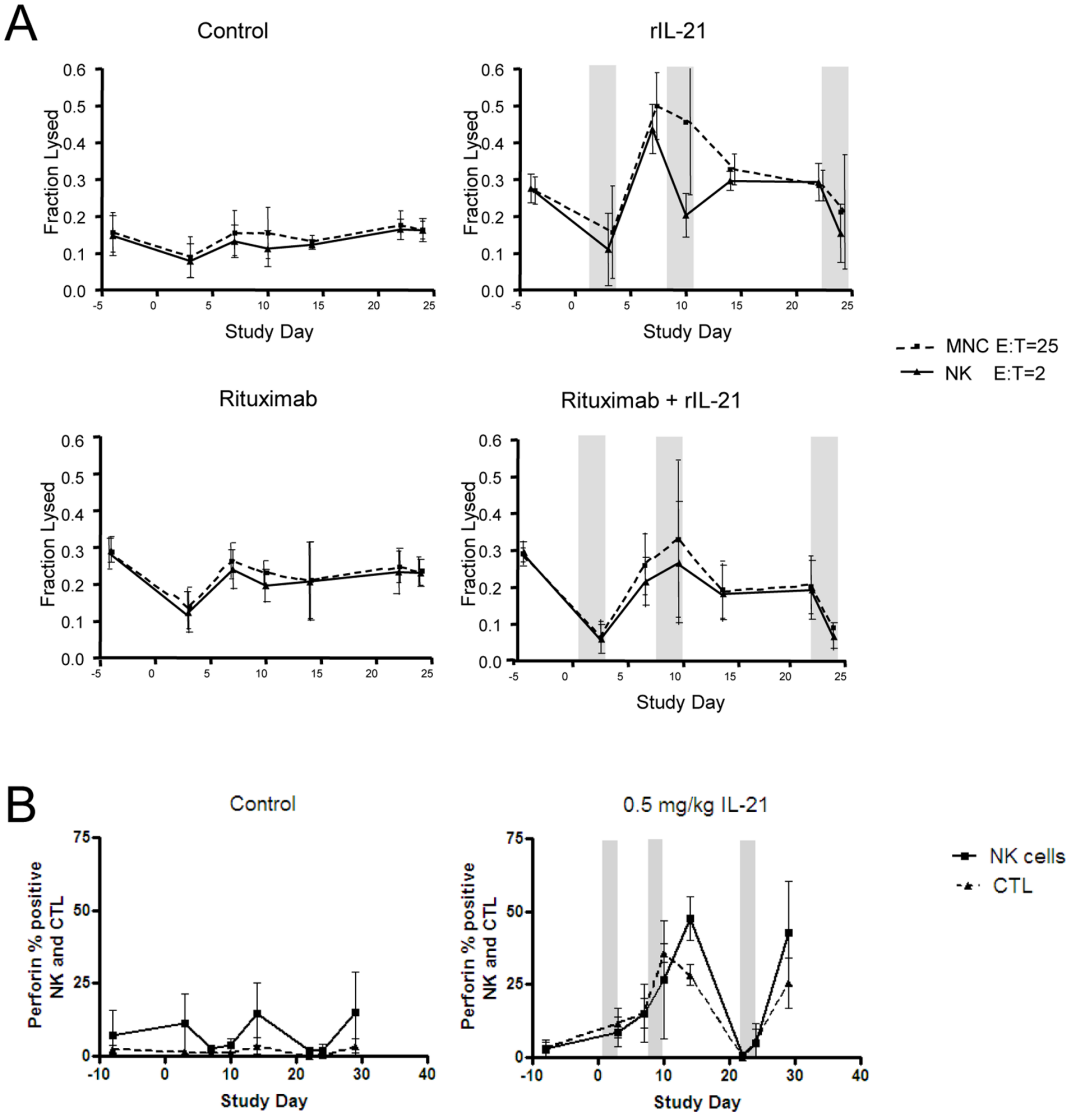
Activation of innate effector cells in cynomolgus monkeys treated with rIL-21. (A) ADCC activity in PBMC preparations in animals treated with vehicle control, weekly rituximab (0.05 mg/kg), rIL-21 (0.5 mg/kg for 3 days), or a combination of rituximab (0.05 mg/kg) and rIL-21 (0.5 mg/kg for 3 days). Gray bars indicate rIL-21 dosing days; rituximab was co-administered before the first rIL-21 dose in each cycle (days 1, 8, and 20). (B) Percentage of peripheral blood NK cells and cytotoxic T lymphocytes (CTL) staining positive for perforin. Gray bars in C indicate dosing days, error bars show standard error of the mean (SEM). (A, B) N = 3 animals per group.

Treatment of monkeys with rIL-21 enhanced the ADCC activity of NK cells (Figure 3A). Following a transient post-treatment lymphopenia and very low ADCC activity (day 3), increased NK activity was observed on days 7 and 10 in rIL-21 treated groups. Fluctuations from baseline occurred in control and rituximab-only treated groups, but these changes were much less dramatic than in rIL-21-treated groups (Figure 3A). Increased perforin content was also observed in NK cells and CTLs (Figure 3B).

**Figure 4:**
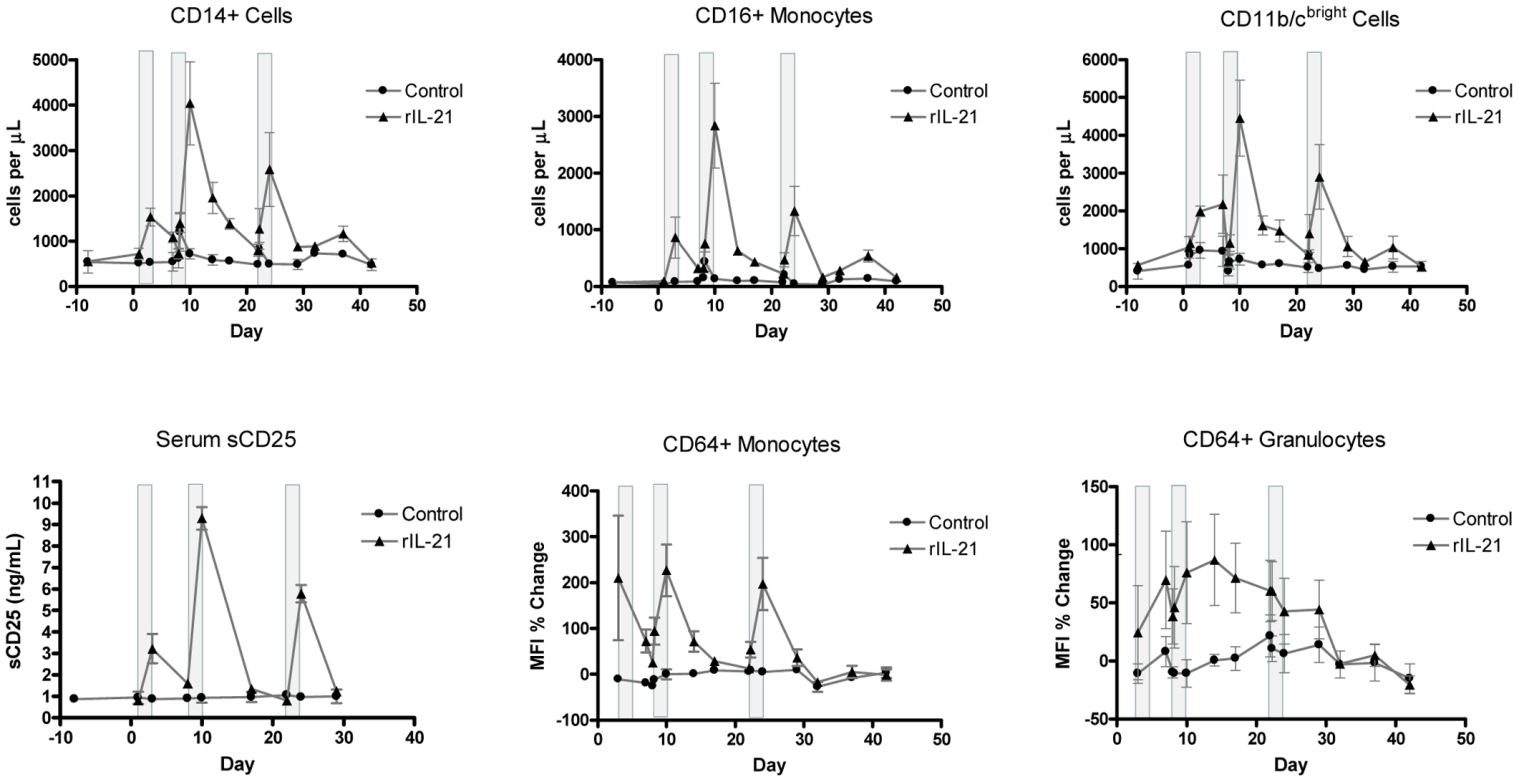
Changes in immune cell markers. Graphs show changes in serum soluble CD25 (sCD25) and CD14+, CD16+, CD11b/c^bright^, and CD64+ cells in peripheral blood of vehicle control group and group treated with rIL-21 alone (0.5 mg/kg, 3 days). E:T = effector to target cell ratio. Dosing with rIL-21 (0.5 mg/kg) was by slow i.v. injection on days 1–3, 8–10 and 22–24. Gray bars indicate rIL-21 dosing days, error bars show SEM. N = 3 animals per group.

Other biomarkers of immune activation noted in rIL-21-treated groups were an increase in peripheral blood monocytes, increases in serum soluble CD25 and increased expression of CD11b, CD11c, CD16 and CD64 on myeloid cells (Figure 4). The dynamics of these changes closely followed the rIL-21 dosing schedule and were mostly resolved before the next dosing cycle.

Depletion of CD20+ B cells in cynomolgus monkeys, whose B cells are functionally similar to human B cells, was used as a model for anti-lymphoma treatment in the preclinical development of rituximab [23]. Cynomolgus monkeys have a characteristic B cell population expressing higher levels of CD20 antigen than human B cells, allowing for dose refinement in this model. While a clinical equivalent dose of 10 mg/kg rituximab depletes all peripheral B cells in cynomolgus monkeys, a dose of 0.05 mg/kg will efficiently deplete CD20^high^ cells without affecting the CD20^low^ population [22], [24], [25].

**Figure 5:**
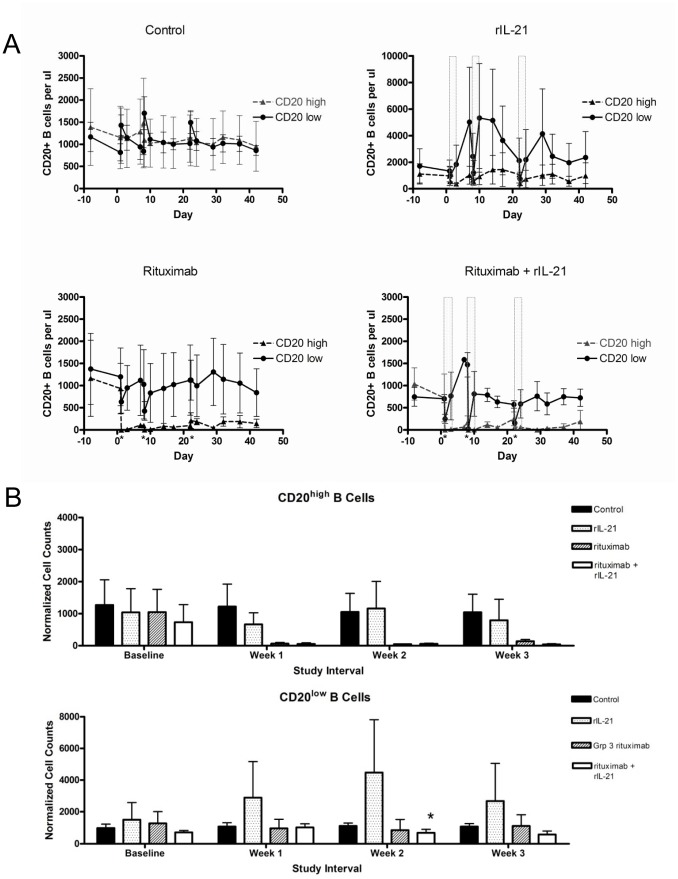
Effects on B cells in cynomolgus monkeys treated with combination rIL-21 and rituximab. The trapezoidal method was used to calculate peripheral cell counts over time (AUC) for the first 7–8 days of each dosing cycle and normalized to a daily average cell count. Graphs show CD20^high^ and CD20^low^ cells in animals treated with vehicle, weekly rituximab (0.05 mg/kg), rIL-21 (0.5 mg/kg for 3 days), or rituximab co-administered before the first rIL-21 dose in each cycle (days 1, 8, and 20). (B) Rituximab in combination with rIL-21 decreased the counts of CD20^low^ B cells, compared to rIL-21 alone, after the second dosing cycle. Open bars indicate rIL-21 dosing days; rituximab was co-administered before the first rIL-21 dose in each cycle (days 1, 8, and 20, indicated by asterix). Note: scales are expanded for control and rituximab-treated groups compared to rIL-21-treated group. (B) Percentage change in normalized CD20^low^ B cell counts after each dosing cycle. (*p<0.05, repeated measures ANOVA comparing combination groups to 0.5 mg/kg rIL-21 group only). Error bars show SEM. (A, B) N = 3 animals/group.

Changes in circulating B cells were observed in all groups except the vehicle control group (Figure 5A). Treatment with low dose rituximab resulted in depletion of the CD20^high^ population and no change in circulating CD20^low^ B cells. Interestingly, treatment with rIL-21 resulted in a strong increase in peripheral B cell counts, especially those with the CD20^low^ phenotype (Figure 5B). The CD20^low^CD21^−^ cells, a population that normally represents a minor fraction in peripheral blood, showed the most dramatic changes. One possible explanation for this observation is that rIL-21 may mobilize B cells from peripheral tissues and transiently increase circulating levels of CD20^low^ cells. Other peripheral lymphocyte populations also responded to rIL-21 treatment with sharp fluctuations. While T cell subsets showed strong increases between dosing cycles, NK cells counts did not rebound above baseline values.

Compared to the rIL-21 treated group, animals treated with low dose rituximab+rIL-21 showed smaller fluctuations in CD20^low^ B cell counts over time, especially in the second dosing cycle (*p*<0.05, Figure 5B). This suggests that rIL-21 treatment improved rituximab efficacy against this normally resistant population.

A second study was performed using the clinical equivalent dose of rituximab (10 mg/kg) in combination with rIL-21 (0.3 or 1.5 mg/kg) once weekly for 4 weeks, followed by a dose-free period of 30 days; no vehicle control or rIL-21 alone control groups were included in this study. Immunophenotyping analysis showed that total, naïve and memory B cells were depleted from blood following rituximab treatment (Figure S1).

**Figure 6:**
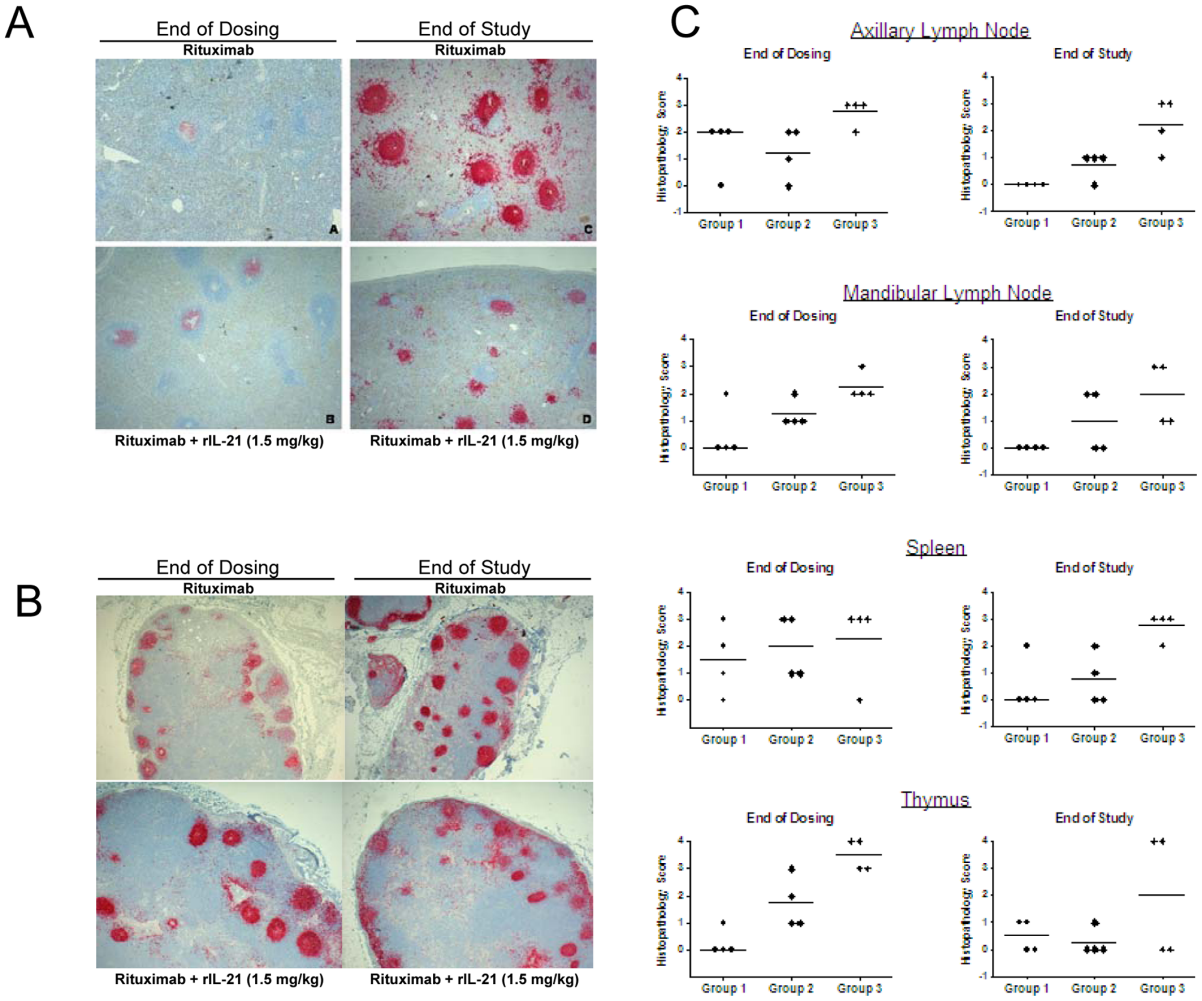
rIL-21 enhances rituximab-mediated B cell depletion in cynomolgus monkeys. B cell depletion in animals treated with 10 mg/kg rituximab (Group 1) and in animals given 10 mg/kg rituximab plus rIL-21 at 0.3 mg/kg (Group 2) or 1.5 mg/kg (Group 3) for four weekly doses, followed by a dose-free period of 30 days. Immunohistochemistry of CD20+ cells in spleen (A) and mandibular lymph node (B) at end of dosing (terminal sacrifice) and end of the study. Images were collected using an Olympus BX51 microscope equipped with a PlanApo 2×/0.08 lens and U-CMAD3 camera, and captured with Colorview and analySIS Soft Imaging System software (Olympus, Tokyo Japan). (C) Lymphoid atrophy scores in lymphoid tissues collected at the end of dosing and at the end of the study. Histopathology scores for severity of atrophy were subjectively assigned as 0, none; 1, minimal; 2, slight; 3, moderate; 4, marked. Bar indicates median group score.

All groups showed dose-related lymphoid atrophy of the germinal center and follicles in the spleens and lymph nodes. Following a 30 day recovery period, small lymphoid follicles and a lack of germinal centers persisted in the 1.5 mg/kg rIL-21+rituximab group. In contrast, lymphoid tissues from animals treated with 0.3 mg/kg rIL-21+rituximab or rituximab alone had developed germinal centers and showed fewer signs of lymphoid atrophy (Figure 6).

Taken together, the monkey studies demonstrate that rIL-21 enhances rituximab-mediated depletion of B cells, inducing a more sustained depletion from tissues and peripheral blood than rituximab given alone.

### rIL-21 has Diverse Actions on B Lymphoma Cell Lines

**Figure 7:**
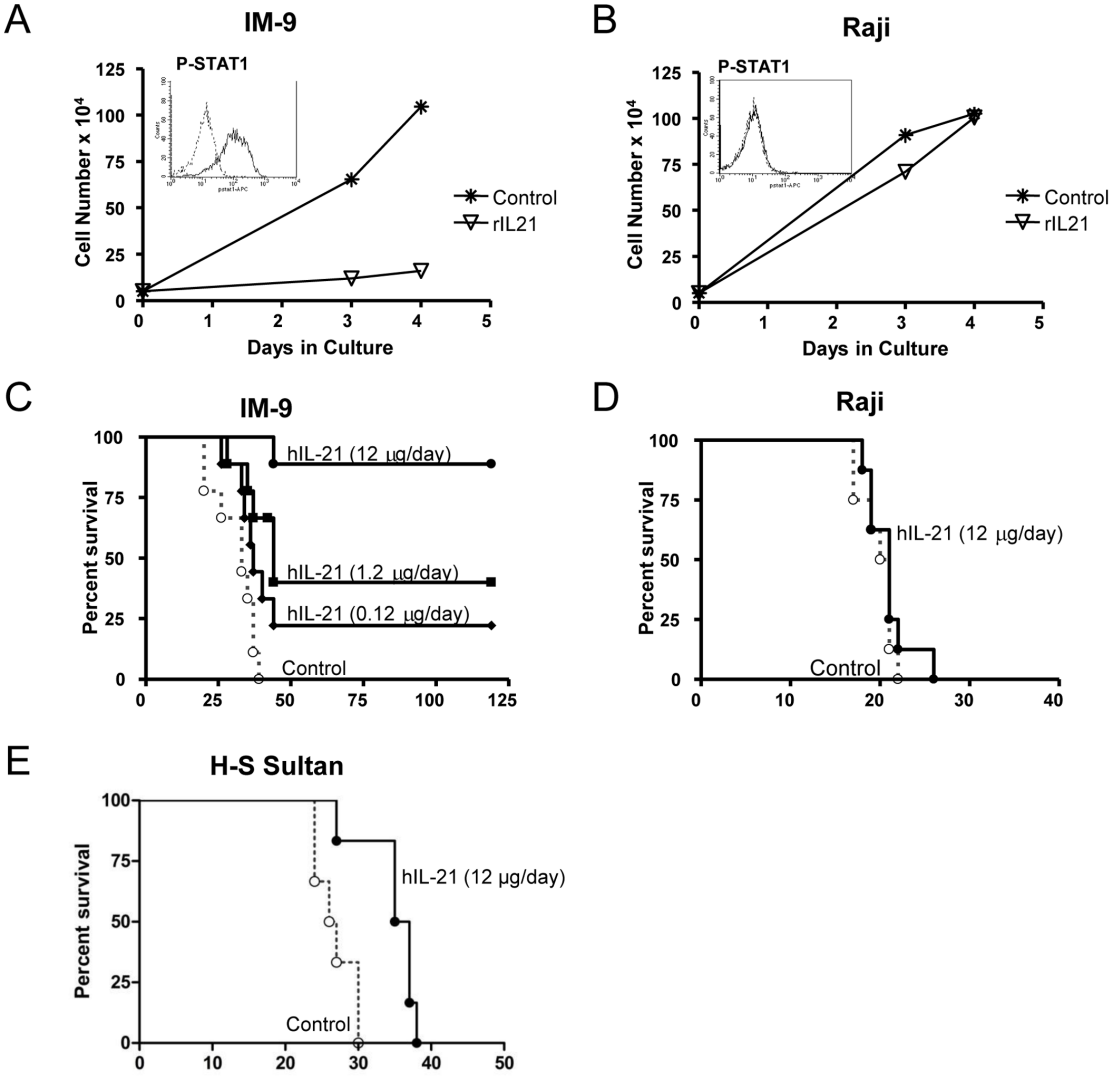
rIL21 induced STAT1 signaling and growth inhibition for IM-9 but not Raji lymphoma cells. Growth curves for (A) IM-9 and (B) Raji cells when cultured with 50 ng/ml of rIL-21 after 3 days pre-treatment under the same conditions. Insets show phospho-STAT1 signaling IM-9 and Raji cells after 15 min culture with 10 ng/ml IL-21. Lower panels show survival curves for SCID mice (6–10/group) injected i.v. with 10^6^ cells of the (C) IM-9, (D) Raji, or (E) H-S Sultan lymphoma lines. Human rIL-21 or control treatment (PBS) was administered by 28-day mini-osmotic pump implanted one day after tumor cell injection.

Eight human B-cell lymphoma cell lines were cultured with rIL-21 to investigate the effects of rIL-21 on cell growth *in vitro*. Compared to media controls, IM-9, WSU-NHL and MC116 were strongly inhibited (∼80%) by rIL-21 after 7 days in culture (Table 2). Growth curve analysis of MC116 showed that initial treatment with rIL-21 caused a modest growth increase at 48 hrs, followed by decreased growth. In rIL-21-responsive lines, pre-treatment with rIL-21 resulted in growth inhibition even if no rIL-21 was included in the subsequent culture. The Raji, DOHH2 and Ramos lines were unaffected by rIL-21 treatment; CESS and HS-Sultan lines had intermediate responses (Table 2, Figures 7A and 7B,).

**Table 2:** IL-21R expression, STAT phosphorylation, and growth inhibition in human B lymphoma lines treated with rIL-21.

Cell Line	IL-21R^1^	CD132^1^	P-STAT3^2^ (SD)	P-STAT1^2^ (SD)	% Growth Inhibition^3^
IM-9	26	60	2.40 (0.07)	6.26 (5.40)	85
WSU-NHL	59	11	4.52 (0.03)	5.79 (4.55)	81
MC116	251	108	1.97 (0.47)	3.13 (0.88)	74
CESS	54	285	1.38 (0.42)	1.49 (0.06)	47
HS-Sultan	37	3	1.47 (0.09)	1.17 (0.23)	33
Raji	39	12	1.14 (0.04)	1.05 (0.06)	3
DOHH2	27	0	1.13 (0.06)	0.95 (0.08)	0
Ramos	56	3	1.89 (0.26)	1.64 (0.66)	0

1 Mean fluorescence intensity (MFI) minus background staining with isotype-matched control antibody.

2 Ratio of MFI for P-STAT staining in stimulated versus control cells after 15 minute treatment with 10 ng/mL rIL-21. Mean and SD are from 2 experiments. Following rIL-21 stimulation, STAT5 phosphorylation was detected in only two cell lines, MC-116 and IM-9.

3 Percentage growth inhibition was determined by live cell counts after 7 days culture with 50 ng/mL rIL-21 or control media (representative data from one of two experiments). Similar results were observed with ^3^H incorporation.

Expression of cognate IL-21R on these cell lines was assessed. All showed moderate to strong staining for IL-21R, but CD132 staining was variable. To evaluate whether the receptor was functional, lymphoma lines were stained for phosphorylation of STAT1 and STAT3 after rIL-21 stimulation. Three cell lines (IM-9, WSU-NHL and MC116) showed robust accumulation of phosphorylated (P)-STAT1 and P-STAT3 following rIL-21 treatment, with mean fluorescence intensity (MFI) ratios of approximately 2 to 6 compared with unstimulated cells. Lower levels of P-STAT1 and P-STAT3 were induced by rIL-21 in the other cell lines. In Ramos cells, rIL-21 caused an 89% increase in P-STAT3 accumulation, but no growth inhibition (Table 2).

Treatment with rIL-21 also inhibited proliferation of certain lymphoma tumor lines in tumor-engrafted SCID mice. Subcutaneous delivery of 1.2 µg rIL-21/day over 28 days prolonged survival of SCID mice bearing IM-9 cells from 33 days to 44 days, and 90% of the mice treated with 12 µg rIL-21/day were alive at day 119 (Figure 7C). In other experiments, rIL-21 prolonged the survival of SCID mice bearing HS-Sultan cells. However, mice bearing Raji cells were not protected by rIL-21 treatment (Figure 7D and 7E). Thus, the growth inhibition *in vivo* (Figure 7) was concordant with *in vitro* results (Table 2), suggesting that rIL-21 may have direct anti-proliferative activity against a subset of lymphomas.

### IL-21 Enhances Rituximab-mediated Survival in Mouse B Lymphoma Models

Based on the above results, we reasoned that IL-21 treatment might also enhance the anti-tumor activity of rituximab *in vivo*. Since rIL-21 (human) does not efficiently stimulate murine IL-21R, murine IL-21 (mIL-21) was used to test combinations with rituximab in disseminated lymphoma models using HS Sultan cells and Raji cells. Murine IL-21 was found to have comparatively little direct cytotoxic activity against these human cell lines *in vitro* (data not shown).

**Figure 8:**
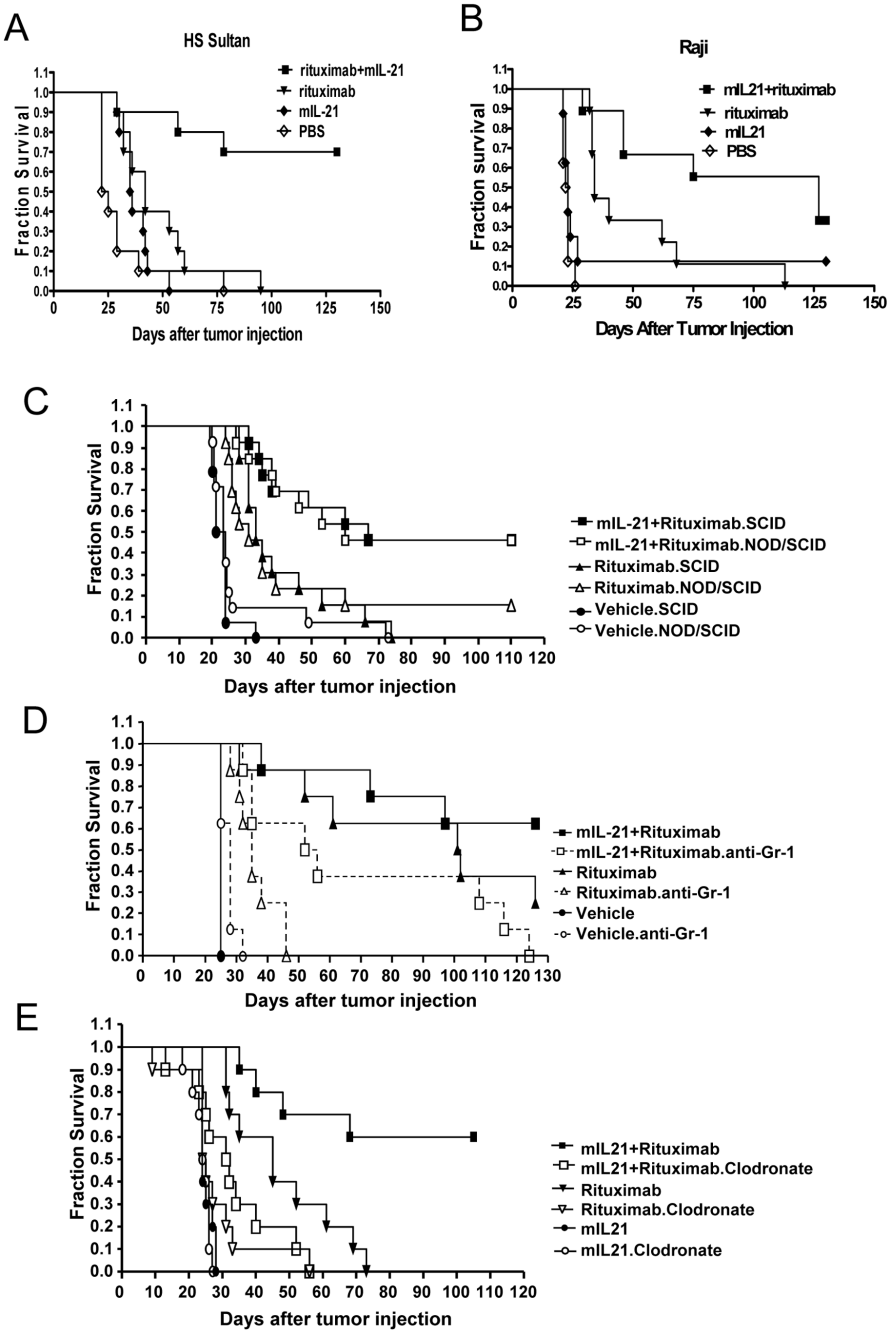
Murine IL-21 plus rituximab prolongs the survival of SCID mice bearing disseminated lymphoma tumor cells. (**A, B**) SCID mice (n = 10/group) were injected i.v. with 10^6^ lymphoma cells and then treated with rituximab alone (20 µg on days 3, 7, 11, 15, 19), mIL-21 alone (100 µg days 1–5), or mIL-21 and rituximab. Significant prolongation of survival was observed in the rituximab plus mIL-21 group when compared to rituximab alone (*p* = 0.0006) in the HS-Sultan model (A). Mice in the Raji model (B) were treated as above, except that mIL-21 was given on days 3–7 and rituximab was given on days 5, 9, 13, 17, and 21. Mice in the combination group survived longer than those treated with rituximab alone (*p* = 0.0079). (**C, D, E**) Test of effector cell function with Raji lymphoma models established in SCID or NOD/SCID mice as described above. (C) NOD/SCID mice with impaired NK cells compared to SCID mice. Equivalent survival times were observed for NOD/SCID and SCID mice given rituximab plus rIL-21 (p = 0.012) or rituximab alone. (D) SCID mice injected i.p. with 50 µg of anti-Gr-1 antibody on day -1, 4, 9 and 14 to deplete granulocytes or with PBS (control). Survival was significantly decreased for depleted mice given rituximab plus rIL-21 (p = 0.012) or rituximab alone (p = 0.001) compared with non-depleted mice. (E) SCID mice injected i.v. with liposomes containing clodronate to deplete macrophages or PBS (control). Survival was significantly decreased for depleted mice given rituximab plus rIL-21 treatment (p = 0.0115) or rituximab alone (p = 0.0011) compared with non-depleted mice.

SCID mice injected with HS Sultan lymphoma cells were given vehicle (phosphate buffered saline (PBS)) alone, mIL-21 alone, rituximab alone, or mIL-21 plus rituximab. Death was delayed in mice treated with mIL-21 or rituximab alone, compared to vehicle treatment (median survival 35.5 and 42.0 days versus 23.5 days), but all mice in these groups succumbed within 100 days (Figure 8A). When mIL-21 and rituximab were combined, 70% of the mice survived more than 125 days and survival was significantly improved compared to rituximab alone (*p* = 0.0006).

Similarly, mice injected with Raji cells were given vehicle alone, mIL-21 alone, rituximab alone, or rituximab+mIL-21. Survival in the mIL-21 group was equivalent to vehicle, with a median survival time of 23 days (Figure 8B). Rituximab extended survival to a median of 34 days (*p*<0.0001). Combination therapy with mIL-21+rituximab extended survival significantly compared to treatment with rituximab alone (median survival 127 days, *p* = 0.0079).

To identify effector mechanisms active in mIL-21+rituximab-mediated survival, SCID mice deficient in or depleted of NK cells, neutrophils, or macrophages were used as hosts for the Raji lymphoma model. To evaluate the role of NK cells, NOD/SCID mice, which display reduced NK cell activity [26], were compared to SCID mice. Both strains were injected with Raji cells and treated with vehicle, rituximab alone or rituximab+mIL-21 (Figure 8C). Equivalent survival was observed in NOD/SCID mice and SCID mice treated with mIL-21+rituximab, with median survivals of 60 and 67 days, respectively, and 45% of the mice surviving long term (110 days). Treatment with rituximab alone yielded median survival times of 30 and 32 days in NOD/SCID and SCID mice, respectively.

Neutrophil depletion with anti-Gr-1 monoclonal antibody significantly reduced survival of SCID mice treated with rituximab plus mIL-21 relative to non-depleted mice (p = 0.0115). No depleted animals survived after 125 days, compared with 62.5% of non-depleted mice (Figure 8D). For mice treated with rituximab alone, neutrophil depletion reduced the median survival time from 102 days to 35 days (P = 0.0011). Neutrophil depletion did not affect median survival time in vehicle-treated mice (28 versus 25 days).

Clodronate liposome treatment was used to deplete macrophages in SCID mice with Raji lymphoma. Macrophage depletion reduced the efficacy of mIL-21+rituximab (Figure 8E). None of the macrophage-depleted mice survived longer than 55 days, whereas 60% of the non-depleted group survived 105 days (*p* = 0.0115). Treatment with rituximab alone resulted in low median survival in macrophage-depleted mice (25 days compared to 45 days for non-depleted mice, *p* = 0.0011). For mice treated with mIL-21 alone, median survival was 24 days for both groups. These results suggest that macrophage depletion *per se* does not influence mouse survival or tumor growth, and that the anti-tumor effects mediated by rituximab+mIL-21 treatment require macrophage activity.

## Discussion

Our data show multiple effects of rIL-21 on immune cells. Human and cynomolgus NK cells had increased capacity for ADCC following culture with rIL-21. Similarly, a recent study using NK cells from patients with B-cell chronic lymphocytic leukemia demonstrated enhanced cytotoxicity and ADCC when the cells were stimulated with IL-21 and rituximab, compared to control or rituximab treatment alone [27]. The current data indicate that, in primates, rIL-21 treatment induces and preserves expression of lytic granules, FcγRIIIa, adhesion molecules and other proteins that support ADCC activity.

In cynomolgus monkeys treated with rIL-21, sharp fluctuations in peripheral blood B lymphocyte counts were observed in relation to rIL-21 dosing. The kinetics of these changes, which included a transient decrease followed by robust post-dosing increase beyond baseline, suggest that rIL-21 treatment may affect lymphocyte recirculation patterns, rather than altering proliferation of these cells. In support of this notion, depletion of B cell zones of lymphoid tissues has been observed shortly after rIL-21 treatment in cynomolgus monkeys [28]. In addition, our *in vitro* studies of human B cells showed that rIL-21 up-regulated ICAM-1 and promoted a more differentiated state.

In our studies of cynomolgus monkeys, B cell depletion was more rapid in peripheral blood and of longer duration in tissues from the animals treated with rIL-21 in combination with the clinical dose of rituximab. Enhanced depletion of CD20^low^ B cells from peripheral blood in groups treated with low-dose (0.05 mg/kg) rituximab was more difficult to demonstrate in the face of rIL-21-induced changes in the peripheral blood compartment. Nevertheless, the rituximab+rIL-21 group showed less pronounced fluctuations in B cells than the animals treated with rIL-21 alone. This difference suggests that rIL-21 improved rituximab activity, since the CD20^low^ cells would not normally be susceptible to rituximab-mediated depletion in the low-dose setting. Humans do not have a circulating CD20^high^ B cell population, and the expression of CD20 on human peripheral blood B cells is similar to that of the CD20^low^ population in cynomolgus monkeys [24]. Studies in human CD20 transgenic mice have shown that lymphoid tissues provide cellular microenvironments that protect against apoptosis, and mobilization of CD20+ cells from these tissues is necessary for rituximab-mediated depletion [29]. Because susceptibility to depletion by rituximab is correlated with the dynamics of cellular recirculation, mobilization of B cells from lymphoid tissues by rIL-21 in humans could result in enhanced depletion by rituximab by increasing access to effector cells and by decreasing resistance to apoptosis following CD20 engagement.

We found that rIL-21 directly inhibits the growth of some B lymphoma lines, and that growth inhibition was correlated with the ability to signal through the IL-21R/γ_C_. The strongest inhibition was observed in cells that displayed activation of both STAT1 and STAT3. The delayed growth inhibition and the extended duration of rIL-21 effects on responsive cell lines suggest that rIL-21 alters cellular programming to induce a less proliferative state. Results of mouse xenogeneic tumor models were concordant with the *in vitro* proliferation data. As mouse innate immune cells respond very poorly to rIL-21, these data suggest a direct inhibitory effect on responsive lymphomas *in vivo*.

As described above, rIL-21 pretreatment of human and cynomolgus monkey NK cells in vitro enhances rituximab-mediated ADCC. Based on these findings, we predicted IL-21 would enhance the antitumor activity of rituximab in mice by promoting NK cell-mediated ADCC. However, we were unable to show in a number of methods a role for mouse NK cells in mIL-21 plus rituximab-mediated rejection of B lymphoma cells in mice. We found mIL-21 enhances rituximab-mediated survival equivalently in SCID mice, NOD/SCID mice (NK-function impaired) and in SCID mice treated with anti-asialo-GM1 antibody to deplete NK cells (Figure 8C and data not shown). Additionally, we were unable to show ex-vivo ADCC or NK cell activity using NK cells from mice bearing lymphoma and treated with rituximab+IL-21 (data not shown). These results are in contrast to a study of rituximab in a xenogeneic lymphoma model which found at least a partial role for mouse NK cells [30]. Data from a Her-2-positive syngeneic mouse tumor model in immunocompetent mice also support a role for mIL-21 stimulating NK cells to produce IFN-γ and enhancing antibody therapy [31], and studies of a Raji lymphoma model with a chimeric anti-CD70 monoclonal antibody also found NK cells important for prolonging survival [32]. Differences in the tumor models and/or the antibodies may explain these discrepancies. Evidence for different mechanisms of action based on antibody isotype and antigen specificity has been observed in mouse models of B cell depletion [33]. Our in vitro/ex vivo results agree with those of others who find mouse NK cells require a costimulus (e.g. IL-15 or poly I/C) to respond to IL-21 [9]. Nevertheless, considering the effects of IL-21 on human and cynomolgus monkey NK cells, it is likely that our mouse model under-represents the potential of IL-21 to enhance rituximab therapy of B lymphoma by activation of NK cells.

Depletion of granulocytes with an anti-Gr-1 antibody reduced the efficacy of rituximab alone, which is consistent with a previous report [30], and reduced the fraction of mice surviving long term after treatment with IL-21 plus rituximab from 67% to 0% (Figure 8D). Although mIL-21 may be acting directly on mouse neutrophils causing them to phagocytose opsonized tumor cells, enhance ADCC, or produce cytotoxic oxygen intermediates, a direct effect of IL-21 on neutrophils is not supported by studies with human neutrophils where IL-21R was not detected and human IL-21 did not modulate neutrophil responses including superoxide production, phagocytosis, chemotaxis and cytokine production [34]. In these studies, IL-21 induced IL-8 production by human macrophages that may have led to neutrophil chemotaxis and activation. We have also been unable to detect IL-21R expression by or IL-21 activation of human neutrophils (data not shown). Our results in SCID mice using anti-Gr-1 antibody may be explained by the expression of Gr-1 on macrophages and monocytes [35], [36]. Depletion of macrophages and monocytes by anti-Gr-1 would not only eliminate an important effector cell for ADCC but also prevent the indirect recruitment of neutrophils as observed by Pelletier et al. [34].

Monocytes or macrophages may be the primary effector cell mediating the antitumor activity of mIL-21 and rituximab in our mouse models. After depletion of these cells with clodronate liposomes, we observed a nearly complete loss of the enhanced antitumor activity displayed by IL-21 and rituximab (Figure 8E). Our results suggest that macrophages play an important role in prolonging survival in these models following combination therapy. Mouse macrophages were required in studies employing anti-CD20 monoclonal antibodies to deplete normal B cells [29], [33], [37], and were also required for prolonging survival in a Raji lymphoma model with an anti-CD70 chimeric monoclonal antibody [32]. Several labs have observed human macrophages, monocytes or dendritic cells to express the IL-21R and to respond to IL-21 [38], [39], [40], and our studies in nonhuman primates indicate that IL-21 can stimulate a dose-dependent increase in the number of activated CD64+ monocytes in the peripheral blood of cynomolgus monkeys.

In conclusion, our studies demonstrate direct effects of rIL-21 on normal B cells and growth inhibition in a subset of lymphoma cell lines. Additionally, rIL-21 enhances NK cell ADCC activity in primates and induces activation of innate immune effector cells. In cynomolgus monkeys, rIL-21 enhanced B cell depletion and prolonged the effects of rituximab. The combination of direct action on B cells and indirect enhancement of ADCC activity through activation of effector cells indicate that rIL-21 may improve the anti-lymphoma efficacy of rituximab. Indeed, durable complete remissions were observed in a small subset of patients in a Phase I clinical trial of rituximab+rIL-21 as therapy for low grade B-cell lymphoproliferative disorders [41].

## Supporting Information

Figure S1 **Effects on total, naïve, and memory B cells in cynomolgus monkeys treated with combination rIL-21 and rituximab.** Depletion of total (A), naïve (B), and memory (C) B cells in cynomolgus monkeys treated with 10 mg/kg rituximab alone and in animals given 10 mg/kg rituximab plus rIL-21 at 0.3 mg/kg or 1.5 mg/kg for four weekly doses, followed by a dose-free period of 30 days. Data points indicate group mean, error bars show standard deviation.
(TIF)
