# Supplementary figures and images for: Interleukin-21 Enhances Rituximab Activity in a Cynomolgus Monkey Model of B Cell Depletion and in Mouse B Cell Lymphoma Models
Source: PLoS One. 2013 Jun 25;8(6):e67256. doi: 10.1371/journal.pone.0067256 (PMC3692496; doi:10.1371/journal.pone.0067256)

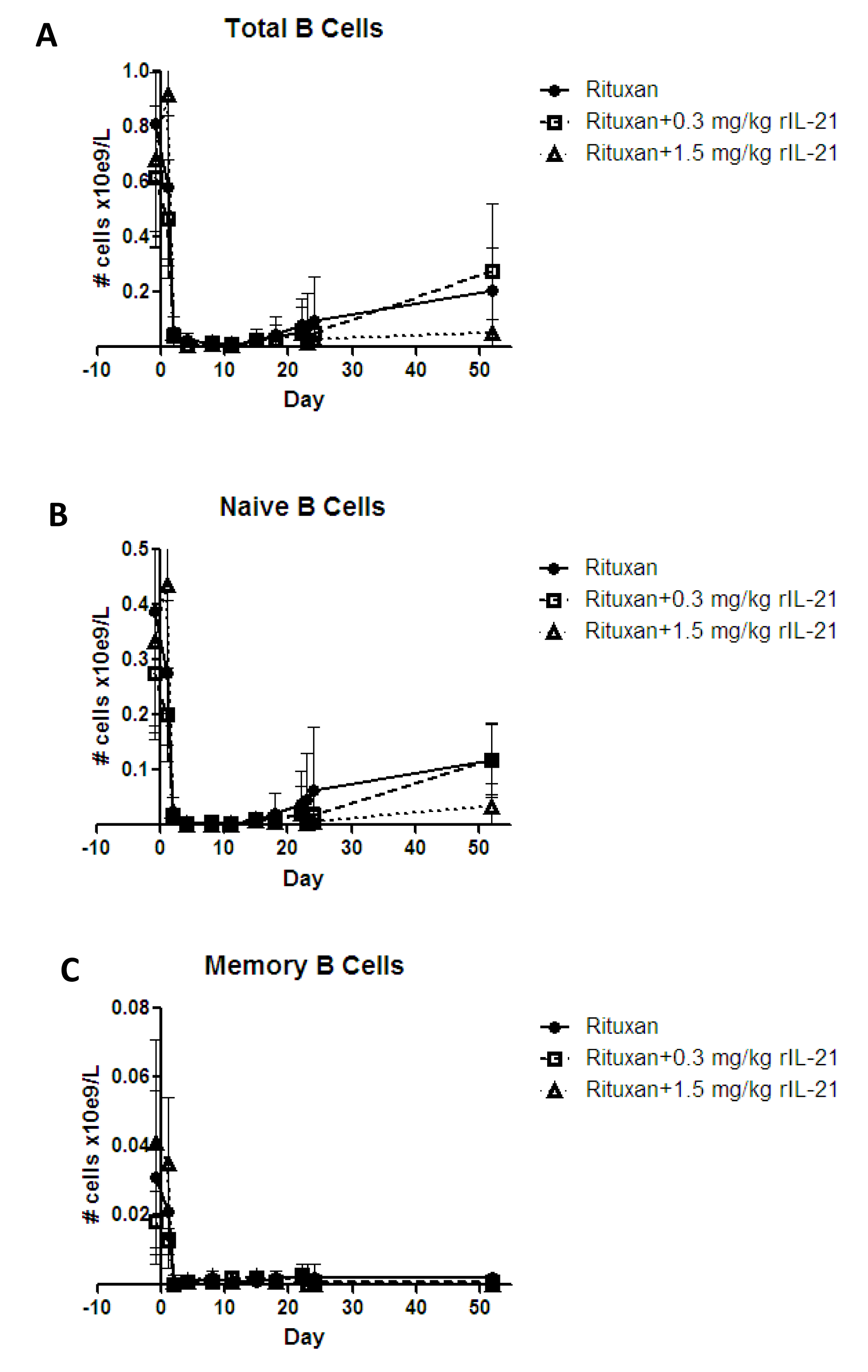

Supplement: Figure S1 — Effects on total, naïve, and memory B cells in cynomolgus monkeys treated with combination rIL-21 and rituximab. Depletion of total (A), naïve (B), and memory (C) B cells in cynomolgus monkeys treated with 10 mg/kg rituximab alone and in animals given 10 mg/kg rituximab plus rIL-21 at 0.3 mg/kg or 1.5 mg/kg for four weekly doses, followed by a dose-free period of 30 days. Data points indicate group mean, error bars show standard deviation. (TIF) [file pone.0067256.s001.tif]
